# The Positive Effect of Perceived Exercise Benefit and the Negative Effect of Perceived Severity of Disease and Weakness on College Students' Amount of Exercise: The Mediate and Suppressor Role of Physical Fitness Evaluation Self-Efficacy

**DOI:** 10.3389/fpsyg.2021.762865

**Published:** 2021-10-28

**Authors:** Li Xue-liu, Xia Mu

**Affiliations:** ^1^College of Physical Education and Health, Nanning Normal University, Nanning, China; ^2^School of Education Science, Guangxi University for Nationalities, Nanning, China

**Keywords:** perceived exercise benefit, perceived severity of disease and weakness, college students, self-efficacy, physical exercise, frequent exercise, motivation factor

## Abstract

**Background:** The decline in the physical fitness of college students has become a serious social problem worldwide. Therefore, exploring the factors affecting the amount of exercise of college students is of great significance in improving college students' physique. According to the expectation value theory and previous studies, perceived exercise benefit and perceived severity of disease and weakness may have positive or negative impact on exercise behavior, and according to the self-efficacy theory, physical evaluation self-efficacy may be the most powerful motivational factors and it play a mediating role between other factors and exercise behavior. Therefore, this study was designed to determine the critical role of physical evaluation self-efficacy in the path of perceived exercise benefit and perceived severity of disease and weakness affecting the amount of exercise of college students.

**Methods:** By means of Physical Fitness Health Belief of College Students Scale and Physical Activity Rating Scale (PARS-3), 801 undergraduate students were investigated in this study.

**Results:** (1) When perceived exercise benefit, exercise self-efficacy, and severity of perceived disease and weakness predicted the amount of exercise separately, the first two have a positive effect on the amount of exercise, but the latter has no effect. However, when these three factors entered the regression equation at the same time, the perceived severity of disease and weakness had a negative effect on the amount of exercise. (2) The influence of physical evaluation self-efficacy on the college students' the amount of exercise was bigger than benefit of perceived exercise and the perceived severity of disease and weakness in both separated and simultaneous comparison conditions. (3) Physical evaluation self-efficacy completely mediated the positive effect of perceived exercise benefits on the amount of exercise and inhibited the negative effect of perceived severity of disease and weakness on the amount of exercise.

**Conclusion:** Physical evaluation self-efficacy has a strong predictive power on the amount of exercise of college students. This was reflected in its ability to mediate the impact of expectation of exercise results and in its ability to suppress the adverse effects of concern about illness on exercise.

## Introduction

College students represent high-quality talent trained by the state. Their healthy growth relates to the future development of a nation. However, the decline in the physical fitness of college students has become a serious global social problem (Li, 2020; Peng et al., 2020). As the bioecological theory of human development (Bronfenbrenner and Morris, 2006; Rosa and Tudge, 2013; Tudge et al., 2016; Xia et al., 2020) predicts, frequent exercise of a certain intensity is a factor that causes good health. Therefore, exploring the factors that affect the amount of exercise that college students engage in is of great significance to improve their physique.

The factors that determine the initiation and maintenance of exercise behaviors are complex. According to the expectation value theory proposed by Vroom (1964), the intensity of motivation required to stimulate and maintain behavior is affected by the specific results produced by the behavior and its value. Based on this theory, if individuals do not expect physical exercise to enhance their physique, or if they do not care about their health despite awareness that exercise will benefit it, they will not engage in physical exercise. This hypothesis is supported by the study of Luo and Du (2017), who found that the perceived exercise benefit dimension (representing the expectation of a result of exercise behavior and its value) in the Physical Fitness Health Belief of College Students scale (Dai et al., 2011) is significantly positively correlated with the amount of exercise. Moreover, Guo and Xu (2011) discovered that the dimensions of life promotion and preventive health care related to physical health in the exercise benefit scale (Sechrist et al., 1987) significantly positively correlate to the exercise action and maintenance stage of urban residents. Exercise may not be initiated solely by expectations for positive results, but also by the fear of negative results. This hypothesis is supported by He and Chen (2016), who reported that teenagers' perceived severity of disease and weakness dimension (representing the fear of disease) in the Physical Fitness Health Belief of College Students scale (Dai et al., 2011) was significantly positively correlated with their amount of exercise. Similarly, Luo and Du (2017) identified a positive relationship between perceived severity of disease and weakness and amount of exercise among junior and senior high school students.

However, evidence supported that perceived exercise benefit and severity of disease and weakness may not affect exercise uniquely or directly. First, studies found that among several motivational factors, individuals' beliefs about their exercise ability—the self-efficacy (Bandura, 1977, 1993, 1997, 2012; Bandura and Cervone, 1983; Bandura and Jourden, 1991) of exercise—can not only affect exercise behavior, but also has the strongest power among many influencing factors. For example, Jin et al. (2018) used both horizontal and vertical comparisons to explore the impact of non-exercise incentives and exercise self-efficacy on college students' autonomous fitness behavior. The results showed that in the horizontal comparison, although both non-exercise incentives and exercise self-efficacy can significantly predict the level of college students' autonomous exercise behavior, the latter has stronger prediction power. In the longitudinal comparison, only exercise self-efficacy and autonomous exercise behavior can predict each other across time. Yu et al. (2021) analyzed the influencing factor of college students' amount of exercise using a structural equation model. The results revealed that among many factors, such as behavior attitude, behavior cognition, teacher support, and peer support, self-efficacy had the strongest predictive power. Second, many activities produce valuable results if consistently implemented. However, if people doubt whether they can succeed, even if they sure the value of the behavior results to themselves, they will not take the initiative to implement the actions (Beck and Lund, 1981; Wheeler, 1983; Dzewaltowski et al., 1990). Third, although there is a lack of research on the relationship between fear of disease and self-efficacy of exercise, other studies found that for patients, self-efficacy is an important factor for them to adhere to exercise. For example, Du and Zhang (2021) reported that general self-efficacy is significantly positively correlated with rehabilitation exercise compliance among patients with a lower limb fracture. Guo et al. (2010) also documented that exercise self-efficacy is significantly positively correlated with exercise participation among college students with serious physical health problems. Intervention studies have consistently found that self-efficacy enhanced by an intervention program can significantly improve rehabilitation exercise behavior among elderly female patients with urinary incontinence (Yang et al., 2013) and elderly diabetic patients (Meng et al., 2011).

**Figure 1:**
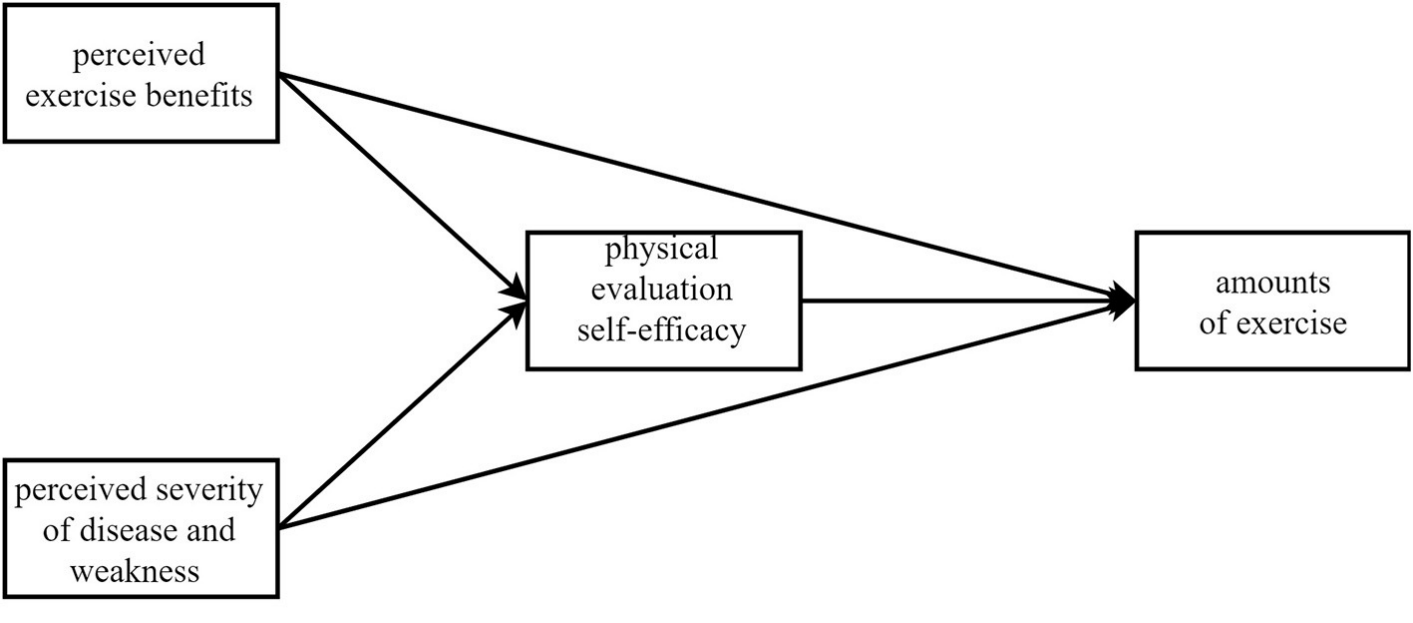
The mediating effect model for Hypothesis 2.

These findings indicate that exercise self-efficacy may be the mediating variable between positive or negative motivation factor and the amount of exercise. Some studies have found that exercise self-efficacy plays a mediating role between other factors and exercise behavior or willingness to exercise. For example, Yang (2016) found that in the youth group, physical exercise self-efficacy partially mediated the impact of social support on physical exercise satisfaction. Furthermore, Wang et al. (2014) found that exercise self-efficacy partially mediated the relationship between leadership behavior and individual exercise persistence in the group. Moreover, Yan et al. (2019) concluded that exercise self-efficacy and physical education (PE) class satisfaction play a complete chain mediation role between college PE teachers' transactional leadership behavior and college students' willingness to adhere to physical exercise. Based on the above research results, three hypotheses can be proposed:

1. The perceived exercise benefit, exercise self-efficacy, and the perceived severity of disease and weakness significantly contribute to the amount of exercise.
2. Among the three factors, the effect of self-efficacy should have strongest power.
3. Exercise self-efficacy mediate the effect of perceived exercise benefit and perceived severity of disease and weakness on the amount of exercise (Figure 1).

Unfortunately, many studies (e.g., Dong, 2017; Luo and Du, 2017; Dong et al., 2018) considered these three factors concurrently as aspects of a higher order factor (i.e., health belief; Dai et al., 2011), rather than exploring them separately. Currently, only one study (Xie, 2013) considers the comprehensive impact of the three dimensions on exercise momentum, separately; however, the relationship hypothesis of variables in Xie's study differs from the current study. In all, the aim of this study is to verify these three hypotheses.

## Materials and Methods

### Research Type and Data Analysis

This quantitative empirical study utilized electronic questionnaires as the method of data collecting, and correlation analysis, regression analysis, and path analysis as the method of data analyzing.

### Participants

College students were selected as the participants. A total of 1,010 students (337 males and 673 females, ranging from 17 to 26 years old, with an average age of 20 years) completed the scales.

### Instruments

#### Measurement of Expectation and Value of Exercise Results

The expectation and value of exercise results were measured using the Perceived Benefits of Exercise subscale from the Physical Fitness Health Belief of College Students scale (Dai et al., 2011). The scale comprised seven items, all of which were graded using a five-point Likert scale, which featured options from one—“completely inconsistent” (one point) to five—“completely consistent” (five points). An example item is as follows: “physical exercise can enhance physical health.” In this study, the internal consistency coefficient of this scale was 0.883.

#### Measurement of Exercise Self-Efficacy

Exercise self-efficacy was measured using the Physical Fitness Evaluation Self-Efficacy subscale from the Physical Fitness Health Belief of College Students scale (Dai et al., 2011). The scale comprised four items, all of which were graded using a five-point Likert scale, which featured options from one—“completely inconsistent” (one point) to five—“completely consistent” (five points). An example item is as follows: “when physical health is poor, I will overcome difficulties to exercise.” In this study, the internal consistency coefficient of this scale was 0.805.

#### Measurement of Perceived Severity of Disease and Weakness

The perceived severity of disease and weakness was measured using its namesake subscale from the Physical Fitness Health Belief of College Students scale (Dai et al., 2011). The scale comprised five items, all of which were graded using a five-point Likert scale, which featured options from one—“completely inconsistent” (one point) to five—“completely consistent” (five points). An example item is as follows: “poor physical health will seriously affect my quality of life.” In this study, the internal consistency coefficient of this scale was 0.854.

#### Measurement of Amount of Exercise

The Physical Activity Rating scale (PARS-3) revised by Liang (1994) was used to measure the students' amount of exercise. The scale evaluates the amount of exercise based on three aspects: intensity, time, and frequency. The specific calculation formula is the amount of exercise = intensity × time × frequency; the highest score is 100 points, and the lowest is zero. Intensity and frequency were measured from “grade 1” (one point) to “grade 5” (five points), and time from “grade 1” (zero point) to “grade 5” (four points). The amount of exercise is divided into three parts. A score of <19 means a small amount of exercise; a score from 20 to 42 represents a medium amount of exercise, and a score of more than or equal to 43 denotes a level of high intensity exercise.

### Procedure

Based on the protection needs of the coronavirus disease 2019 (COVID-19), all surveys were completed online. A link to the scales was sent to the students' online social network group (i.e., QQ group or Wechat group) of the institutes to which the researcher or his collaborators belong. Students were asked to complete the questionnaire voluntarily and all at once. To ensure the quality of the online questionnaire, the significance of the real data to the researchers is emphasized in the questionnaire guide. Moreover, it emphasizes that the whole questionnaire was answered anonymously. In addition, to ensure that the participants responded to the questions carefully, a careless response detection was inserted in the middle of the scales: please choose “not very suitable” for this question. Any students who did not respond to this item were considered to be careless participants and their data were excluded in the subsequent analysis. It took about 5 minutes to complete the questionnaire. Each participant gave their informed consent on the first page of the scales. This study was approved by the school's Institutional Review Board.

## Results

### Effective Participation

Participants who did not respond appropriately to the careless response detection test option, were excluded; the remaining 801 (242 boys) valid questionnaires were available, with an effective recovery rate of 79%.

### Common Method Deviation

The study used a self-report method to collect data; hence, there may be common method deviations (Du et al., 2005). After data collection, the Harman single factor test was used to diagnose the common method deviation. The results show that the eigenvalues of the four factors are >1 without rotation, and the variation of the first factor is 37.75%, which is less than the critical standard of 40%. Therefore, there were no serious common method deviations in this study.

### Descriptive Statistics

According to Table 1, the average score of perceived exercise benefits of college students was 24.14, which was close to the full score of 30, and there was a significant positive correlation between perceived exercise benefits and the other three variables, and they were all at the high significance level of 0.01; The average score of college students' physical fitness evaluation self-efficacy was 12.48, which was above the median of 10, and there was a significant positive correlation between the self-efficacy of physical evaluation and the other three variables, and they were all at the high significance level of 0.01; the average score of perceived severity of disease and weakness was 17.02, which was above the median of 12.5. There was a significant positive correlation between the perceived severity of disease and weakness, perceived exercise benefits, and physical fitness evaluation self-efficacy, and they were all at a high significance level of 0.01, but there was no significant correlation between the perceived severity of disease and weakness, and the amount of exercise. The amount of exercise for college students was 19.60. According to the judgment standard proposed by Liang (1994), the results demonstrate that the amount of exercise for contemporary college students is small.

**Table 1:** The mean, standard deviation, and correlation of each variable.

	**M**	**SD**	**1**	**2**	**3**	**4**
1. Perceived exercise benefits	24.14	4.03	/			
2. Self-efficacy of physical evaluation	12.48	3.39	0.569^**^	/		
3. Perceived severity of disease and weakness	17.02	4.24	0.545^**^	0.518^**^	/	
4. Amounts of exercise	19.60	19.63	0.144^**^	0.263^**^	−0.03	/

*p < 0.05,

** p < 0.01, ***p < 0.001.

### Verifying the Relative Predictive Power of the Three Factors

To test hypothesis 1, by considering the perceived benefits of exercise, self-efficacy of physical evaluation, and perceived severity of disease and weakness as independent variables X, and by selecting the amount of exercise as the dependent variable, three regression analyses were run. The results are shown in Table 2 for perceived exercise benefits: [*F*_(1, 799)_ = 16.878, *p* < 0.001, *R*^2^ = 0.021], physical fitness evaluation self-efficacy [*F*_(1, 799)_ = 59.41, *p* < 0.001, *R*^2^ = 0.069], and perceived severity of disease and weakness [*F*_(1, 799)_ = 0.674, *p* = 0.412, *R*^2^ = 0.001]. When perceived exercise benefits and physical fitness evaluation self-efficacy enter the regression equation alone, they can explain about 2 and 7% of the variation in exercise amount, respectively, which are positive predictions. The perceived severity of disease and weakness did not, independently, significantly predict the amount of exercise.

**Table 2:** Separate regression analysis results of amount of exercise on three factors.

	***F***	***R^**2**^***	***B***	***se***	***Beta***	***t***
Constant a			2.692	4.173	/	0.645
Perceived exercise benefits	16.878	0.021	0.700	0.170	0.144	4.108^***^
Constant b			0.589	2.556	/	0.230
Self-efficacy of physical evaluation	59.41	0.069	1.523	0.198	0.263	7.708^***^
Constant c			21.891	2.870	/	7.627^***^
Perceived severity of disease and weakness	0.674	0.001	−0.134	0.164	−0.029	−0.821

Constant a is a constant that perceived exercise benefits as an independent value, constant b is self-efficacy of physical evaluation as an independent value, and constant c is the perceived severity of disease and weakness as an independent value. *p < 0.05, **p < 0.01,

*** *p < 0.001*.

Second, the perceived benefits of exercise, self-efficacy of physical evaluation, perceived severity of disease, and weakness were used as predictive variables, and the amounts of exercise were used as outcome variables. The forced entry method was used for regression analysis. The results demonstrated that when perceived benefits of exercise, self-efficacy of physical evaluation, and perceived severity of disease and weakness entered the regression model at the same time, the equation was established [*F*_(7, 797)_ = 33.137, *p* < 0.001, *R*^2^ = 0.111], and the results are shown in Table 3. All of them had a significant predictive effect on exercise level (all *p* < 0.05), which can explain about 11% of the variation in exercise level. The perceived benefits of exercise and self-efficacy in physical evaluation played a positive predictive role, and the perceived severity of disease and weakness played a negative predictive role. Consistent with Hypothesis 1, the predictive power of physical fitness evaluation self-efficacy (standard regression coefficient) was the largest.

**Table 3:** Multiple regression analysis results of amount of exercise on three factors.

	***B***	***se***	***Beta***	***t***
Constant	4.574	4.024	/	1.137
Perceived exercise benefits	0.418	0.212	0.086	1.974^*^
Self-efficacy of physical evaluation	2.006	0.247	0.346	8.133^***^
Perceived severity of disease and weakness	−1.181	0.193	−0.255	−6.109^***^

* p < 0.05, **p < 0.01,

*** *p < 0.001*.

### Verifying the Mediate Effect of Self-Efficacy

**Figure 2:**
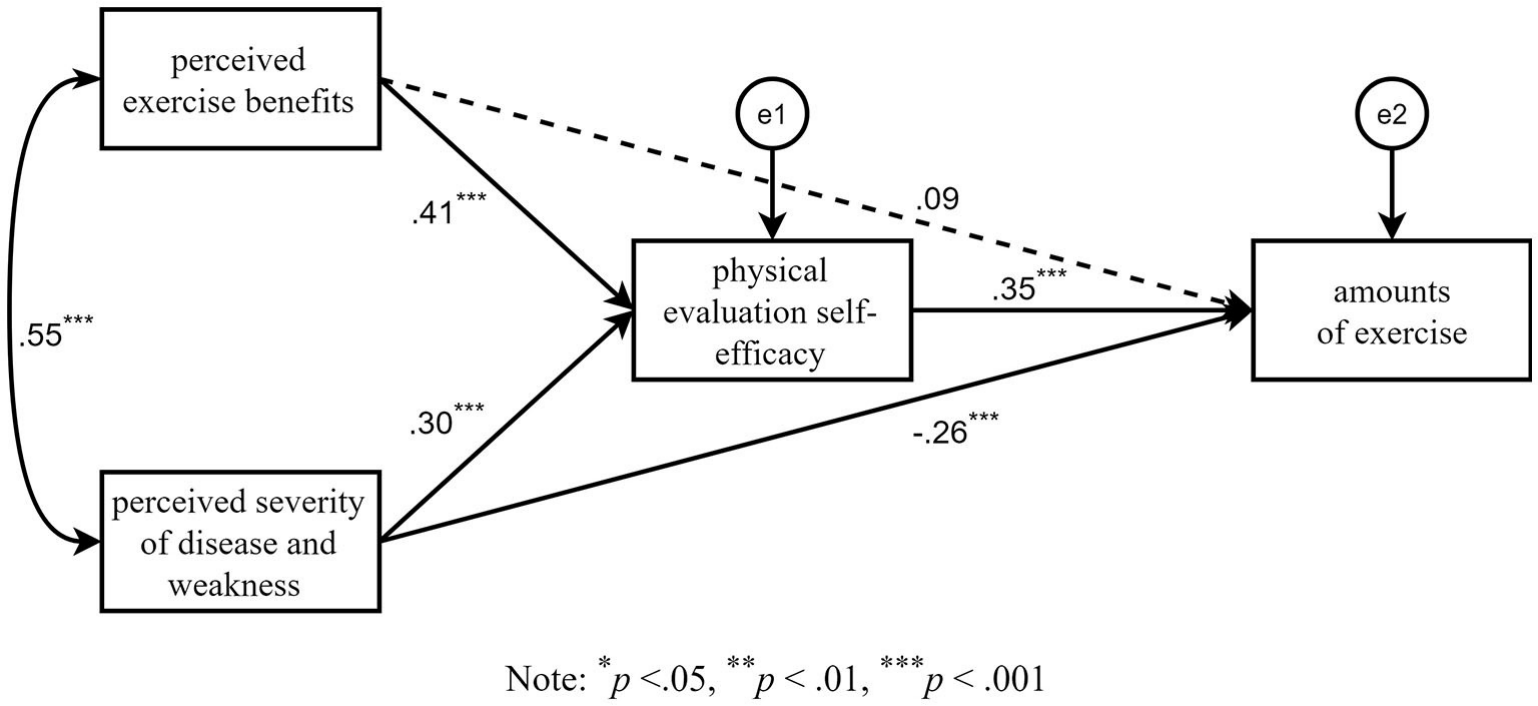
The standard path model of three factors affecting the amount of exercise.

To test hypothesis 3, a path model was established with perceived benefits of exercise, severity of disease, and weakness as independent variables, physical fitness evaluation self-efficacy as mediation variables, and amount of exercise as dependent variables. Amos 24.0 was used for data analysis, and the bootstrap method after bias correction was used to test whether each path effect was significant. A total of 5,000 samples were taken, and the confidence interval was set to 95%. As shown in Table 4, for a single path, the 95% confidence interval of the path of perceived exercise benefit, the amount of exercise has a zero value, so the effect was not significant (*p* = 0.078), except that all other 95% confidence interval paths, whether direct or indirect, did not contain a zero value, so the effect was significant. In addition, because the direct effect of perceived exercise benefit was not significant, it shows that the physical fitness evaluation self-efficacy plays a complete mediating role between perceived exercise benefits and the amount of exercise. However, the directions of indirect and direct effect of perceived severity of disease and weakness (the path seven and eight in Table 4) are opposite. When direct effect and indirect effect move in opposite directions, it means that there is a potential suppression effect by the mediating variable (i.e., physical fitness evaluation self-efficacy) on the independent variable (i.e., perceived severity of disease and weakness) (Wen and Ye, 2014). The final path model is presented in Figure 2.

**Table 4:** Summary of path effect.

**Path**	***B***	***Beta***	**Bias corrected**		***p***
			**95% CI**		
			**Lower**	**Upper**	
1. Self-efficacy of physical evaluation–amount of exercise	2.006	0.350	1.498	2.539	^***^
2. Perceived exercise benefits–self-efficacy of physical evaluation	0.343	0.410	0.283	0.399	^***^
3. Perceived exercise benefits–self-efficacy of physical evaluation–amount of exercise	0.689	0.144	0.483	0.923	^***^
4. Perceived exercise benefits–amount of exercise (direct effect)	0.418	0.090	−0.045	0.879	0.078
5. Perceived exercise benefits–amount of exercise (total effect)	1.106	0.234	0.675	1.555	^***^
6. Perceived severity of disease and weakness–self-efficacy of physical evaluation	0.236	0.300	0.176	0.297	^***^
7. Perceived severity of disease and weakness–self-efficacy of physical evaluation–amount of exercise	0.474	0.105	0.330	0.667	^***^
8. Perceived severity of disease and weakness–amount of exercise (direct effect)	−1.181	−0.260	−1.600	−0.770	^***^
9. Perceived severity of disease and weakness–amount of exercise (total effect)	−0.707	−0.155	−1.111	−0.301	^**^

*p < 0.05,

** *p < 0.01*,

*** *p < 0.001*.

## Discussion

The purpose of this study was to explore three hypotheses: (1) The positive motivation factor (i.e., perceived exercise benefit or exercise self-efficacy) and negative motivation factor (i.e., the perceived severity of disease and weakness) have a significant influence on the amount of exercise. (2) If the influence of the three factors is considered simultaneously, the effect of self-efficacy should be the strongest on the volume of exercise by college students. (3) The influence of positive and negative exercise motivation on college students' amount of exercise is mediated by exercise self-efficacy.

First, Hypothesis 1 was partly verified. On the one hand, consistent with the expectation value and self-efficacy theories, perceived exercise benefit and exercise self-efficacy have a positive correlation with college students' amount of exercise, which again proves the expectation of result, the value of this result and self-efficacy are important positive motivation factors (Vroom, 1964; Bandura, 1977; Maddux, 1995; Xia and Li, 2021). On the other hand, the negative motivation factor (i.e., the perceived severity of disease and weakness) had no relationship with the amount of exercise college students engaged in. Further analysis demonstrated that when perceived exercise benefit, exercise self-efficacy, and perceived severity of disease and weakness entered the regression equation at the same time, the perceived severity of disease and weakness had a negative effect on the amount of exercise. This implies that the real and final influence of the perceived severity of disease and weakness on the amount of exercise is negative, and this effect may be suppressed by the other variable. This result was not consistent with past research (He and Chen, 2016; Luo and Du, 2017), which found a positive correlation between the perceived severity of disease and weakness and the amount of exercise. The reason for this difference may be the different research objects: in the past two studies, the participants were all middle school students, while in this study, the research participants were college students. Compared with middle school students, college students may be more aware of the possibility of a vicious circle between physical weakness and sports; that is, the weaker the physique, the likelier they are to experience physical injury during physical exercise (Song and Cui, 2018; Yang, 2019), which further aggravates their physiques' fragility. Therefore, college students with low evaluations of their physique will actively avoid sports. Moreover, even though both college students and middle school students in China are worried and afraid of getting sick, compared to middle school students, college students have less academic pressure, and so their fitness regime can be more flexible; hence, the demand for intensive exercise is not so strong. Furthermore, Chinese high school students face Gao Kao (i.e., college entrance examination) (Feng et al., 2021; Li, 2021), so they cannot reduce their study time or increase their rest time to maintain their health, like college students can. The only choice they may have is to exercise. This may also explain the results. Future research can further explore whether there are differences regarding the awareness of physical injury, or the choice of health-promoting behavior, between college students and high school students, to further verify this hypothesis.

Second, Hypothesis 2 was fully verified, that is, whether predicting the amount of exercise by college students alone or at the same time, the influence of college students' physical fitness evaluation self-efficacy (standard regression coefficient) is greater than the benefit of perceived exercise, and the perceived severity of disease and weakness. This result is consistent with previous studies (Jin et al., 2018; Yu et al., 2021) that self-efficacy plays a core role in many behavior-influencing factors. Even when simultaneously compared to other motivating factors, the influence of self-efficacy is still stronger.

Finally, Hypothesis 3 was verified. First, after controlling for physical fitness evaluation self-efficacy, the direct impact of the perceived exercise benefits on amount of exercise, is no longer significant, which shows that the impact of perceived exercise benefits on the amount of exercise is completely realized through self-efficacy of physical evaluation. The more certain the causal relationship between exercise and physical health, the more confident college students are in their ability to adhere to exercise, which will enable them to overcome various difficulties and actively initiate various exercise behaviors for a long time. It is worth noting that in previous studies, self-efficacy mostly plays a partial mediating role (e.g., Wang et al., 2014; Yang, 2016) between other factors and exercise behavior, and the complete mediating effect found in this study is relatively rare. This finding has a certain guiding significance for future intervention research, that is, those intervention programs that originally intended to improve their exercise level by enhancing the belief that exercise leads to physical health, can be further improved to directly enhance college students' exercise self-efficacy.

In addition, multiple regressions demonstrated that perceived severity of disease and weakness became significant predictors of amount of exercise only when the model included physical fitness evaluation self-efficacy. This finding indicates that physical fitness evaluation self-efficacy is a suppressor variable (MacKinnon et al., 2000, 2002; Shrout and Bolger, 2002; MacKinnon, 2008; Wen and Ye, 2014) to perceived severity of disease and weakness. Path analysis (including Bootstrap analysis) further demonstrated detail of the suppressing effects—perceived severity of disease and weakness had a significant negative affect on the amount of exercise, but a positive effect on physical fitness evaluation self-efficacy, which had a significant positive affect on the amount of exercise. This implies that disease concerns have two distinct influences on motivation to exercise: (1) it may increase concern of getting hurt while exercising (Song and Cui, 2018; Yang, 2019), thus diminishing motivation to exercise or (2) it can enhance belief in one's ability to maintain exercise which enhances motivation to exercise. This result is partially consistent with the findings that the stronger the self-efficacy of sick people, the more they exercise (Guo et al., 2010; Meng et al., 2011; Yang et al., 2013; Du and Zhang, 2021).

In addition, the negative effect (direct effect) of perceived severity of disease and weakness was suppressed by its positive effect (indirect effect) through the effect of physical fitness evaluation self-efficacy, although its total effect was still negative and significant. According to MacKinnon et al. (2000), this indicates that a stronger negative mechanism remains between perceived severity of disease and weakness and amount of exercise, which has thus far not been included in research. This finding suggests that significant disease concerns can diminish motivation to exercise, but that fear of physical injury can be overcome to some extent by college students who have great confidence in their ability to adhere to exercise. In summary, as reported by previous studies, self-efficacy's strong influence as a motivation factor on behavior has been fully reflected in the field of exercise behavior explored in this study.

This study's primary limitation is that it utilized online rather than offline questionnaires. Due to the absence of the researcher, it was difficult to effectively control the process of completing the questionnaire when submitted online (Yu et al., 2019). A 21% careless response rate in this research also proved that individuals can be careless when completing online questionnaires. However, with the advent of the post-COVID-19 era, online surveys seem to be an inevitable trend. Thus, future research should actively develop and adopt a way to enhance the quality of online questionnaire completion. Perhaps participants' involvement motivation could be improved through external rewards (e.g., offer a monetary reward or lucky draw after completing the questionnaire).

## Data Availability Statement

The raw data supporting the conclusions of this article will be made available by the authors, without undue reservation.

## Ethics Statement

The studies involving human participants were reviewed and approved by Institutional Review Board of Nanning Normal University. The patients/participants provided their written informed consent to participate in this study.

## Author Contributions

LX-l was responsible for the conception, writing of general concepts, and analysing data for this paper. XM was responsible for suggesting revision to the concept and writing style of the paper. All authors contributed to the article and approved the submitted version.

## Funding

This article was supported by the Guangxi Natural Science Foundation Mechanism research of the fluctuation of self-efficacy caused by positive or negative feedback in middle school students' English learning (Grant No. 2018GXNSFBA050037).

## Conflict of Interest

The authors declare that the research was conducted in the absence of any commercial or financial relationships that could be construed as a potential conflict of interest.

## Publisher's Note

All claims expressed in this article are solely those of the authors and do not necessarily represent those of their affiliated organizations, or those of the publisher, the editors and the reviewers. Any product that may be evaluated in this article, or claim that may be made by its manufacturer, is not guaranteed or endorsed by the publisher.
